# Construction of a survival nomogram for gastric cancer based on the cancer genome atlas of m6A-related genes

**DOI:** 10.3389/fgene.2022.936658

**Published:** 2022-08-05

**Authors:** Xiaokang Wang, Kexin Xu, Xueyi Liao, Jiaoyu Rao, Kaiyuan Huang, Jianlin Gao, Gengrui Xu, Dengchuan Wang

**Affiliations:** ^1^ Department of Pharmacy, Shenzhen Longhua District Central Hospital, Shenzhen, China; ^2^ Department of Clinical Medicine, School of the Second Clinical Medicine, Anhui Medical University, Hefei, China; ^3^ Shenzhen Key Laboratory of Respiratory Diseases, Shenzhen People’s Hospital (The First Affiliated Hospital, Southern University of Science and Technology), Shenzhen, China

**Keywords:** gastric cancer, m6A, prognosis, nomogram, TCGA

## Abstract

**Objective:** Based on TCGA database, a prediction model for 1-, 3-, and 5-year overall survival rates of gastric cancer (GC) patients was constructed by analyzing the critical risk factors affecting the prognosis of gastric cancer patients.

**Method:** Clinicopathological features as well as gene signature of GC patients were obtained from TCGA database. Patients were randomly divided into a training cohort and an internal validation cohort. Independent predictors of GC prognosis were analyzed by univariate and multivariate Cox analyses to construct nomogram. The accuracy and reliability of the model was further validated by calibration curves, ROC curves, and C-indexes, and the clinical utility of the model was analyzed by decision analysis curves.

**Result:** Age, sex, N stage, M stage, METTL16, RBM15, FMR1, IGFBP1, and FTO were significantly associated with the prognosis of GC patients, and these predictors were further included in the construction of nomogram. The C-indexes for the training cohort and validation set were 0.735 and 0.688, respectively. The results of the ROC curve analysis indicated that the area under the curve (AUC) exceeded 0.6 in training and validation sets at 1, 3, and 5 years.

**Conclusion:** We have constructed and validated a nomogram that provides individual survival condition prediction for GC patients. The prognostic model integrating gene signatures and clinicopathological characteristics would help clinicians determine the prognosis of patients with GC and develop individualized treatment plans.

## Introduction

Gastric cancer (GC) is one of the most common, aggressive malignancies worldwide, with over 1 million newly diagnosed cases annually, and persists to be the third leading cause of cancer-related mortality (Bray et al., 2018; Siegel et al., 2019). Accordingly, early diagnosis of gastric cancer directly determines the clinical outcomes of patients with GC. It is reported that less than 30% of patients with a diagnosis of gastric cancer survived more than 5 years (Ferlay et al., 2015). However, owing to the silent and occult characteristics of early-stage GC, patients are frequently diagnosed at the advanced stage. Several risk factors contribute to GC, such as age, high salt intake, low fruit and vegetable consumption, and *Helicobacter pylori* infection (Tsugane and Sasazuki, 2007). Among factors mentioned earlier, *H. pylori* plays a pivotal role in predisposing one to chronic gastritis, atrophic gastritis, intestinal metaplasia, dysplasia, and eventually gastric cancer (den Hoed and Kuipers, 2016). Up to now, surgical resection is the best treatment option for patients with gastric cancer. Although there were advances in surgical techniques, radiotherapy, chemotherapy, and neoadjuvant therapy, GC’s overall disease and death burden unacceptably continued to increase (Tan, 2019). Therefore, there is an urgent need to develop an effective strategy for the early diagnosis and treatment of GC.

RNA modification plays an indispensable role in the post-transcriptional regulation of gene expression (He, 2010). Numerous studies have shown that dysregulation of m6A regulator expression and genetic changes are associated with the disruption of a variety of biological processes, including dysregulation of cell death and proliferation, developmental defects, malignancy progression, and abnormal immune regulation (Fu et al., 2014; Pinello et al., 2018; Tong et al., 2018). In addition, a growing number of research suggest that m6A modification is responsible for a variety of human cancers, particularly gastric cancer (Jaffrey and Kharas, 2017). Zhang et al. (2020a) recently found that m6A modifications play an integral role in the development of tumor microenvironment (TME) diversity and complexity, and evaluating the pattern of m6A modifications in individual tumors will help guide more effective immunotherapeutic strategies. Both Wang et al. (2020) and Liu et al. (2022) demonstrated that m6A RNA modification mediated by methyltransferase 3 (METTL3) promoted the growth of gastric cancer. As such, we assessed the clinical correlation of m6A modification and identified the pathways and phenotypes regulated by m6A modification to explore the mechanism of m6A in gastric cancer in this study.

The tumor lymph node metastasis (TNM) staging system, proposed by the American Joint Committee on Cancer (AJCC) and the International Union Against Cancer (UICC), is widely used to evaluate tumor staging and predict the prognosis of cancer patients (Sano et al., 2017). Nevertheless, the TNM staging system is not adequate to precisely predict survival outcomes for individual patients, as other clinicopathological parameters such as age, gender, and ethnicity also affect the ultimate survival rate of GC patients (Ashktorab et al., 2017; Yao et al., 2020). In addition, Huo et al. (2021) recently presented a study investigating the prognostic significance of several genes for GC. Although the study was substantial and the data were analyzed retrospectively, clinical data that play an indispensable role in predicting the prognosis of GC were not covered in their study. Therefore, the nomogram has emerged as a more advanced method due to its ability to predict the survival outcome of an individual based on more comprehensive set of patient characteristics.

In this study, we obtained the clinical data and m6A-related gene expression data provided by TCGA database. We retrospectively analyzed all of them for potential risk factors for GC and then constructed a predictive nomogram to assess the probability of survival at 1, 3, and 5 years.

## Materials and methods

### Data collection

**FIGURE 1:**
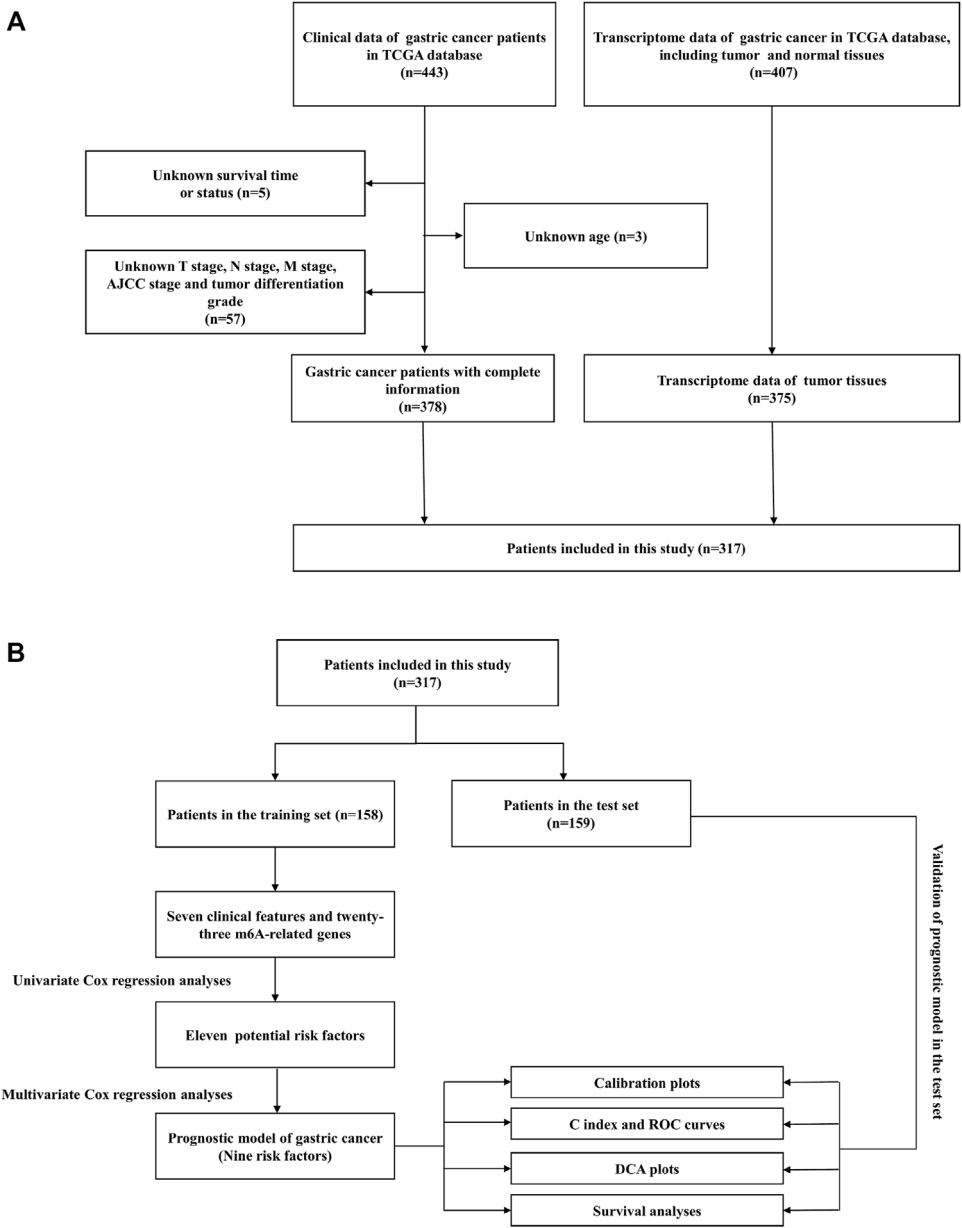
**(A)** Flowchart of the patient screening process. **(B)** Flowchart of the research process of this article.

This study investigated the prognosis of GC patients using the Cancer Genome Atlas (TCGA) database (https://portal.gdc.cancer.gov/). TCGA database is a collaboration between the National Cancer Institute (NCI) and the National Human Genome Research Institute (NHGRI) of the United States project to provide a large, free reference for cancer research by collecting and organizing a variety of histological data related to cancer. In this study, the transcriptome profiling for the GC project was selected in the format of HTSeq-FPKM, which included 32 normal tissue samples and 375 cancer tissue samples. Clinical data for the “bcr xml” workflow type were also downloaded for 443 GC patients. Patients with missing clinical data will be excluded from subsequent analyses. Figure 1A illustrated the screening process for this study.

### Variables and outcomes

The variables initially included in this study were divided into two categories, including 23 m6A-related genes and several clinical variables. Based on previous studies, m6A-associated genes were classified into three categories, involving writes (METTL3, METTL14, METTL16, WTAP, VIRMA, ZC3H13, RBM15, and RBM15B), readers (YTHDC1, YTHDC2, YTHDF1, YTHDF2, YTHDF3, HNRNPC, FMR1, LRPPRC, HNRNPA2B1, IGFBP1, IGFBP2, IGFBP3, and RBMX), and erasers (FTO and ALKBH5). Clinical variables extracted from TCGA database were listed as follows: age, sex, tumor differentiation grade, AJCC stage, T stage, M stage, N stage, survival time, and survival status. The outcome was overall survival (OS), which was defined as the time from initiation to death due to any cause. For subjects lost to follow-up before death, the time of the last follow-up visit was usually considered as the time of death.

### Statistical analysis

After setting the seed number, patients were divided into training set and validation set at a ratio of 1:1.

### Univariate Cox analysis and multivariate Cox regression for independent prognostic factors

Univariate Cox analysis was performed to determine the association between survival outcomes and variables. These variables included demographic characteristics (age and gender), tumor characteristics (grade, AJCC stage, T stage, M stage, and N stage), and 23 m6A-related genes. Statistically significant potential risk factors were further analyzed by multivariate Cox regression, and hazard ratios (HRs) with 95% confidence intervals (CIs) were calculated. Considering that the AJCC stage is a comprehensive result of T, M, and N stages, the AJCC stage is not considered as one of the variables in the multivariate regression analysis.

### Construction of the prognostic nomogram

According to the results of multivariate regression analyses, clinical characteristics and m6A-related genes were integrated through the “rms,” “foreign,” “survival,” and “regplot” packages of R software to construct prognostic nomogram. Scores for each risk factor listed in the nomogram were summed to predict OS at 1, 3, and 5 years for individual GC patients. Simultaneously, we grouped GC patients into low-risk groups and high-risk groups according to median scores. Survival analyses were performed between the high-risk and low-risk groups.

### Validation of the prognostic nomogram

Discrimination is measured by the area under the curve (AUC) of the ROC curve and the concordance index (C-index). Ranging from 0.5 to 1, excellent discrimination is represented by values of 0.6 or more for both AUC and C-index. Furthermore, we also used the calibration plot to measure the extent of proximity between predicted risk and actual risk. Decision curve analysis (DCA) also was used to determine whether the predictive nomogram can improve the prognosis of GC patients in the clinical decisions.

## Results

### Characteristics of the gastric cancer patients

A total of 317 patients with GC were obtained from TCGA database. Fifty percent of the patients from TCGA database were used as the training cohort and the remaining patients were treated as the internal validation cohort. The detailed selection process and study process are presented in Figure 1. In the training set, men accounted for 70% of the total number of patients, 6.94% patients were in M1 stage, and 20.8% patients were in N3 stage, while in the validation cohort, 61.1% were men, 4.21% patients were in M1 stage, and 17.9% patients were in N3 stage. The detailed clinicopathological characteristics of the patients are displayed in Table 1.

**TABLE 1:** Clinicopathological characteristics in GC patients.

	Training cohort (*N* = 158)	Validation cohort (*N* = 159)	*p* Value
Status, n (n%)			1.000
Alive	95 (60.1)	96 (60.4)	
Dead	63 (39.9)	63 (39.6)	
Age	66.7 (10.9)	64.0 (10.2)	0.021
Gender, n (n%)			0.007
Female	72 (45.6)	48 (30.2)	
Male	86 (54.4)	111 (69.8)	
Grade, n (n%)			0.036
Grade_I	5 (3.16)	2 (1.26)	
Grade_II	63 (39.9)	45 (28.3)	
Grade_III	90 (57.0)	112 (70.4)	
Stage, n (n%)			0.008
Stage_I	29 (18.4)	13 (8.18)	
Stage_II	40 (25.3)	61 (38.4)	
Stage_III	68 (43.0)	71 (44.7)	
Stage_IV	21 (13.3)	14 (8.81)	
T, n (n%)			0.256
T1	10 (6.33)	5 (3.14)	
T2	33 (20.9)	30 (18.9)	
T3	68 (43.0)	84 (52.8)	
T4	47 (29.7)	40 (25.2)	
M, n (n%)			0.263
M0	144 (91.1)	151 (95.0)	
M1	14 (8.86)	8 (5.03)	
N, n (n%)			0.639
N0	52 (32.9)	47 (29.6)	
N1	40 (25.3)	43 (27.0)	
N2	37 (23.4)	32 (20.1)	
N3	29 (18.4)	37 (23.3)	
METTL3	4.09 (1.49)	4.20 (1.60)	0.534
METTL14	2.67 (0.73)	2.72 (0.84)	0.616
METTL16	3.72 (1.05)	3.94 (1.33)	0.102
WTAP	10.9 (2.82)	11.4 (2.98)	0.193
VIRMA	6.38 (2.50)	6.22 (1.99)	0.529
ZC3H13	14.6 (6.96)	14.6 (6.57)	0.940
RBM15	4.42 (1.56)	4.59 (1.63)	0.332
RBM15B	12.9 (3.79)	13.2 (4.09)	0.556
YTHDC1	9.35 (2.05)	9.69 (2.18)	0.149
YTHDC2	3.22 (1.37)	3.20 (1.45)	0.912
YTHDF1	26.5 (11.3)	27.5 (10.0)	0.376
YTHDF2	20.3 (4.90)	20.3 (5.48)	0.973
YTHDF3	17.5 (5.93)	17.4 (5.72)	0.831
HNRNPC	38.7 (9.88)	40.0 (10.7)	0.255
FMR1	5.61 (2.77)	6.07 (2.82)	0.150
LRPPRC	20.6 (8.99)	21.1 (9.22)	0.620
HNRNPA2B1	85.8 (24.6)	90.6 (28.9)	0.114
IGFBP1	1.34 (4.69)	1.81 (8.35)	0.542
IGFBP2	38.4 (50.3)	45.4 (61.3)	0.268
IGFBP3	55.1 (46.8)	52.7 (46.1)	0.633
RBMX	29.6 (8.38)	29.0 (7.34)	0.522
FTO	3.39 (1.20)	3.70 (1.37)	0.035
ALKBH5	22.9 (7.41)	23.3 (7.60)	0.645

### Construction of the prognostic nomogram

**FIGURE 2:**
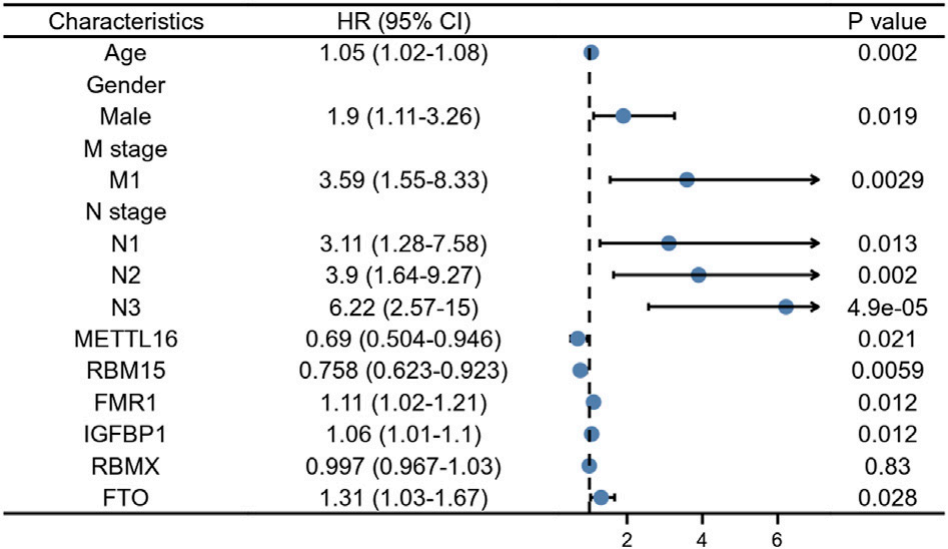
Forest plot for multivariate Cox regression analysis of GC patients in the training cohort.

**FIGURE 3:**
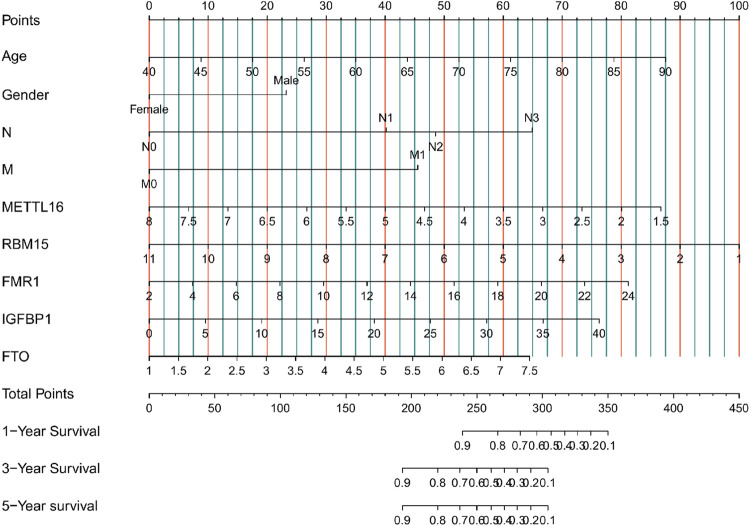
Nomogram for predicting 1-, 3-, and 5-year overall survival (OS) for GC patients in the training cohort.

At a *p*-value cutoff of 0.2 for univariate Cox analysis and multivariate Cox analysis, the potential risk factors were listed as follows: age, gender, M stage, N stage, and five m6A-related genes (METTL16, RBM15, FMR1, IGFBP1, and FTO) (Figure 2). We performed a nomogram using independent variables associated with OS identified in the training set. The nomogram sums the scores for each risk factor identified on the scale, and by adding up the scores for individual items in the nomogram, the 1-, 3-, and 5-year OS for an individual patient can be predicted from the total scores shown at the bottom of the graph (Figure 3). The results of univariate and multivariate Cox analyses are presented as HRs and corresponding 95% CIs in Table 2.

**TABLE 2:** Univariate and multivariate analyses of training cohort.

Characteristic	Univariate analysis hazard rate (95% CI)	*p* Value	Multivariate analysis hazard rate (95% CI)	*p* Value
Age	1.02 (0.99–1.04)	0.189	1.05 (1.02–1.08)	0.002
Gender				
Female	Reference			
Male	1.68 (1–2.82)	0.05	1.9 (1.11–3.26)	0.019
Grade				
Grade_I	Reference			
Grade_II	26442913.45 (0–Inf)	0.995		
Grade_III	25956327.49 (0–Inf)	0.995		
Stage				
Stage_I	Reference			
Stage_II	2.09 (0.74–5.88)	0.161		
Stage_III	2.62 (1.02–6.74)	0.046		
Stage_IV	6.29 (2.26–17.48)	0		
T				
T1	Reference			
T2	73938849.45 (0–Inf)	0.996		
T3	76637295.08 (0–Inf)	0.996		
T4	71683544.93 (0–Inf)	0.996		
M				
M0	Reference			
M1	2.42 (1.15–5.1)	0.02	3.59 (1.55–8.33)	0.0029
N				
N0	Reference			
N1	2.36 (1.08–5.17)	0.031	3.11 (1.28–7.58)	0.013
N2	2.4 (1.1–5.25)	0.028	3.9 (1.64–9.27)	0.002
N3	3.77 (1.75–8.13)	0.001	6.22 (2.57–15)	0.000049
METTL3	0.96 (0.82–1.13)	0.625		
METTL14	0.85 (0.6–1.21)	0.374		
METTL16	0.82 (0.63–1.06)	0.131	0.69 (0.504–0.946)	0.021
WTAP	1 (0.92–1.09)	0.959		
VIRMA	1.02 (0.92–1.12)	0.748		
ZC3H13	0.98 (0.95–1.02)	0.293		
RBM15	0.87 (0.74–1.03)	0.113	0.758 (0.623–0.923)	0.0059
RBM15B	0.99 (0.93–1.06)	0.872		
YTHDC1	1.01 (0.89–1.13)	0.919		
YTHDC2	0.97 (0.8–1.18)	0.782		
YTHDF1	1 (0.99–1.02)	0.661		
YTHDF2	0.98 (0.94–1.03)	0.511		
YTHDF3	0.99 (0.95–1.03)	0.675		
HNRNPC	1.01 (0.98–1.03)	0.557		
FMR1	1.09 (1.01–1.18)	0.035	1.11 (1.02–1.21)	0.012
LRPPRC	1 (0.97–1.03)	0.885		
HNRNPA2B1	1 (0.99–1.01)	0.544		
IGFBP1	1.03 (1–1.07)	0.069	1.06 (1.01–1.1)	0.012
IGFBP2	1 (1–1.01)	0.685		
IGFBP3	1 (1–1.01)	0.288		
RBMX	1.02 (0.99–1.05)	0.136	0.997 (0.967–1.03)	0.83
FTO	1.14 (0.94–1.38)	0.171	1.31 (1.03–1.67)	0.028
ALKBH5	0.98 (0.94–1.01)	0.214		

### Calibration and validation of the prognostic nomogram

**FIGURE 4:**
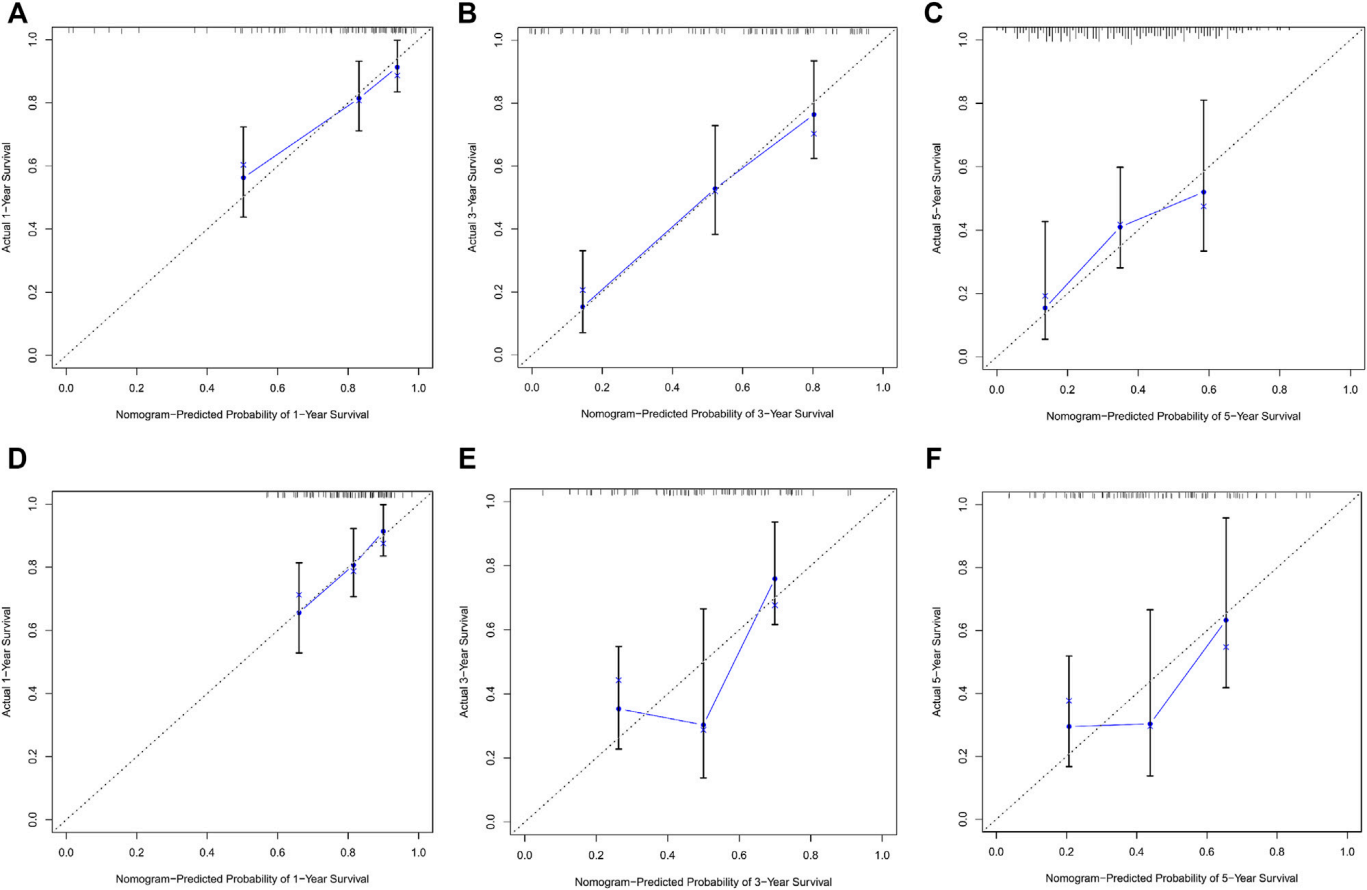
**(A–C)** Nomogram calibration plots to predict 1-, 3-, and 5-year overall survival (OS) in the training cohort. **(D–F)** Nomogram calibration plots to predict 1-, 3-, and 5-year overall survival (OS) in the validation cohort.

**FIGURE 5:**
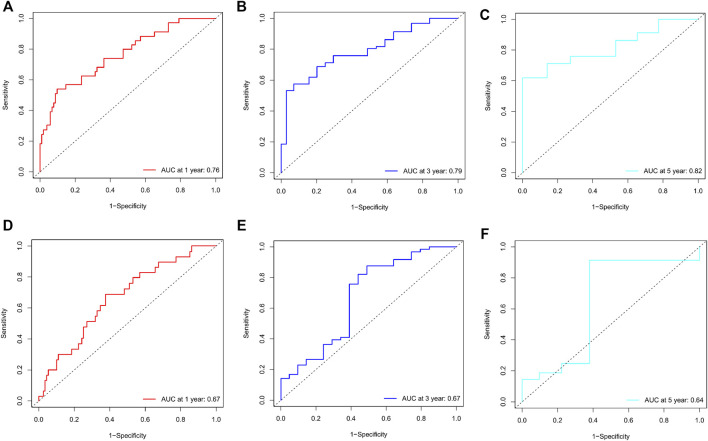
**(A–C)** Nomogram ROC curves to predict 1-, 3-, and 5-year overall survival (OS) in the training cohort. **(D–F)** Nomogram ROC curves to predict 1-, 3-, and 5-year overall survival (OS) in the validation cohort.

**FIGURE 6:**
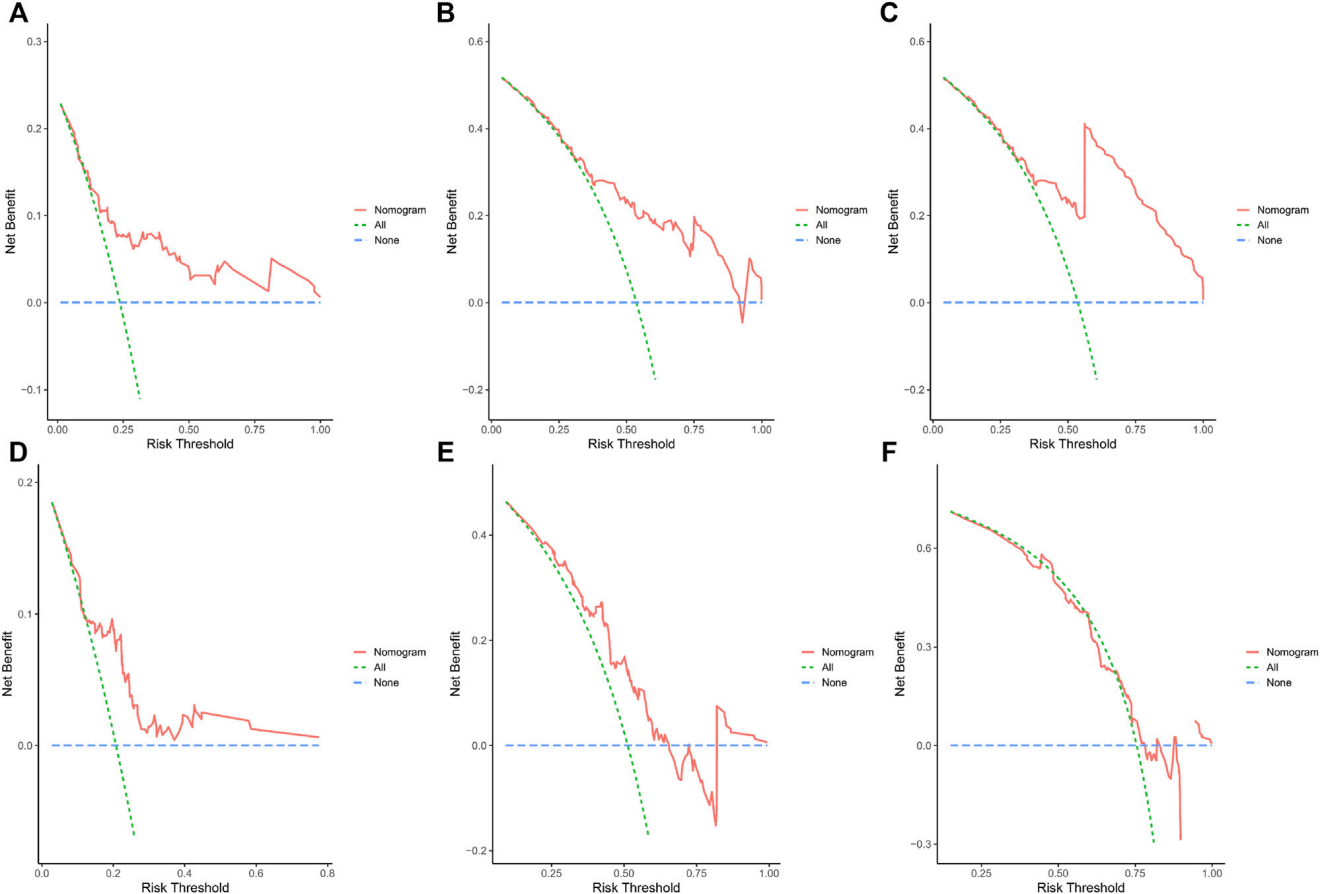
**(A–C)** DCA predicting 1-, 3-, and 5-year OS in the training cohort. **(D–F)** DCA analysis predicting 1-, 3-, and 5-year OS in the validation cohort.

Calibration curves illustrated that there is a strong agreement between predicted survival probabilities and actual survival outcomes (Figure 4). The C-index values for GC patients were 0.735 (95% CI = 0.668–0.802) and 0.688 (95% CI = 0.614–0.762) for training cohort and internal validation cohort, respectively, which demonstrated excellent discrimination. We also plotted the ROC curves and calculated the corresponding AUC values. The AUC values for predicting OS at 1, 3, and 5 years were 0.76, 0.79, and 0.82 in the training cohort and 0.67, 0.67, and 0.64 in the validation cohort, respectively (Figure 5). The AUC values were greater than 0.6, which revealed an excellent accuracy of this predictive model. In addition, as presented in Figure 6, the DCA also indicated excellent clinical applicability for predicting the overall survival of GC patients.

Incorporating the results of the DCA curve, C-index, ROC curve, and calibration curve, we found that the prediction model constructed based on the abovementioned factors had a significant predictive value for OS in GC patients with high accuracy and clinical applicability.

### Survival analyses

**FIGURE 7:**
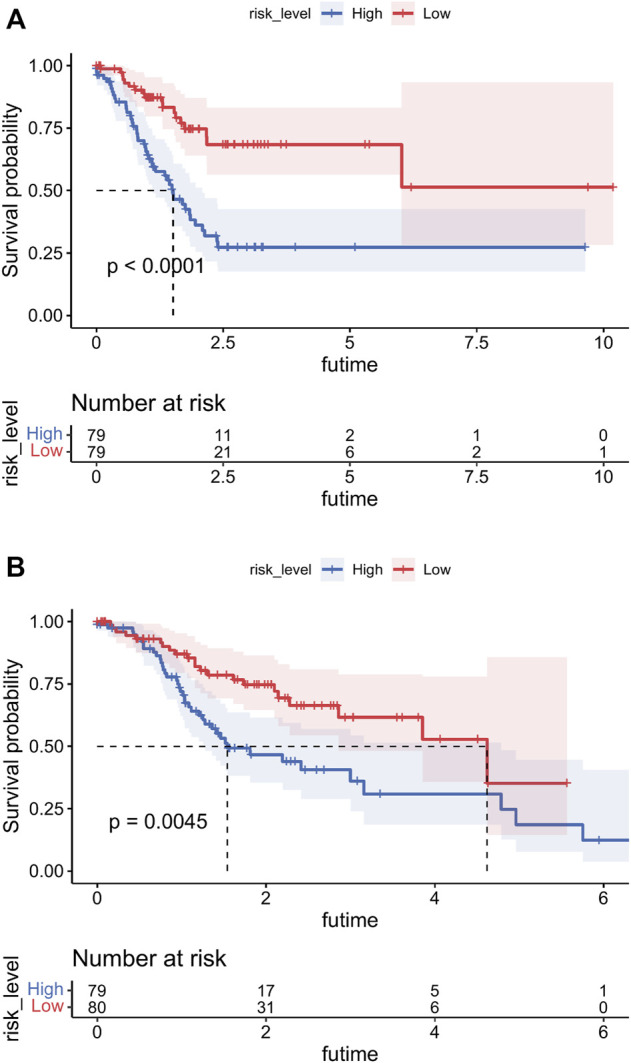
Overall survival (OS) Kaplan–Meier curves for patients in the low- and high-risk groups. **(A)** Training cohort. **(B)** Internal validation cohort.

After summing the risk scores of all independent risk factors, patients were divided into low-risk group and high-risk group according to the median score. Considering all prognostic factors and the different risk groups, survival analyses were performed by Kaplan–Meier plots for both the training and internal validation groups (Figure 7). The prognosis was better in the low-risk group than in the high-risk group (*p* < 0.05).

## Discussion

Gastric cancer represents the second most common cause of cancer (Joshi and Badgwell, 2021). GC is a multifactorial disease, with both environmental and genetic factors influencing its onset and development (Machlowska et al., 2020). Therefore, the prognosis of GC is of great importance to patients. Currently, although there is a large amount of constructed models to predict the prognosis of GC patients, the variables included were confined, especially the m6A-related genes they incorporated were limited, and other important clinicopathological characteristics such as age, gender, and staging were ignored (Su et al., 2019; Zhang B. et al., 2020; Li et al., 2021; Yue et al., 2022). The AJCC staging system is still the most widely used method to assess the prognosis of patients with GC based on factors such as the location of the primary lesion, tumor size, and the presence of distant metastases (Kumagai and Sano, 2021). However, it has some drawbacks that should not be overlooked. It ignores other important clinicopathological characteristics of patients that can affect survival, such as gender, age, ethnicity, as well as cancer genes involved in the formation and development of tumor cells. Genes have been shown to play a remarkably significant role in the initiation, growth, progression, metastasis, immune evasion, and suppression of many kinds of cancer (Dhanasekaran et al., 2022; Luo et al., 2022). A number of gene-specific studies have been carried out to predict the prognosis of tumors, particularly aggressive malignancies, focusing on ferroptosis, pyroptosis, and m6A-related genes (Ghosh et al., 2022; Liang et al., 2021; Zhou Z. et al., 2022). N6-methyladenosine (m6A) RNA modification has emerged as a novel regulatory mechanism for controlling eukaryotic gene expression. m6A is the most abundant internal RNA modification in eukaryotic cells and has received increasing attention in recent years (Sun et al., 2019; Huang et al., 2020). m6A regulates gene expression and cellular processes, including cellular self-renewal, differentiation, invasion, and apoptosis (He et al., 2019). The mechanisms of m6A RNA modification under physiological and pathological conditions have also been revealed. m6A modifications are modified by m6A methyltransferases or writers, such as METTL3/14/16, RBM15/15B, ZC3H3, VIRMA, CBLL1, WTAP, and KIAA1429, and removed by demethylases or erasers, including FTO and ALKBH5. It is recognized by the m6A binding proteins YTHDF1/2/3, YTHDC1/2, IGF2BP1/2/3, and HNRNPA2B1, which are also known as “readers” (Jiang et al., 2021). Most studies have shown that m6A modifications can influence the complexity of cancer progression by regulating biological functions associated with cancer (Ma et al., 2019). The role of m6A modifications in the development, metastasis, and invasion of gastric cancer has been gradually elucidated as potential mechanisms have been revealed. As such, m6A and its regulators contribute to the prognosis of GC, and are expected to be targeted for cancer diagnosis and treatment (Zhao et al., 2021; Hu et al., 2022). Currently, many studies have constructed prognostic models for patients with gastric cancer-targeting m6A modifications (Athauda et al., 2020; Wang et al., 2022). However, a failure to integrate m6A-related genes with other critical clinicopathological characteristics implied that their predictive models had unmitigated limitations.

This study was based on TCGA database, and screened for genetic and detailed clinicopathological characteristics in the tissues of 317 GC patients. Fifty percent of GC patients were used for modeling, with the remaining patients in the internal validation cohort. After univariate Cox regression and multivariate Cox regression, we derived that patient age, sex, N stage, M stage, METTL16, RBM15, FMR1, IGFBP1, and FTO were independent prognostic factors for OS in GC patients and constructed predictive models. The results of our study are consistent with the AJCC grading results. Patients with GC have a worse prognosis when the extent of their primary tumor is more extensive and when there are lymph node metastases and distant metastases. Notably, the extent of the primary tumor and depth of invasion were statistically significantly associated with adverse prognosis for patients with gastric cancer (N1: HR = 3.15, 95% CI 1.28–7.73; N2: HR = 4.05, 95% CI 1.71–9.95; N3: HR = 6.58, 95% CI 2.74–15.8). Our results showed that men had a worse prognosis than women. In line with our findings, Athauda et al. (2020) showed that women with gastric cancer had a more favorable clinical outcome than men. Wang et al. (2021) recently revealed that METTL16-mediated m6A methylation promotes GC cell proliferation through enhanced cyclin D1 expression, and both *in vitro* and *in vivo* experiments confirmed that METTL16 promotes the growth of the tumor by GC cell proliferation. Consistent with our study, other studies have similarly shown that RBM15 is associated with the prediction of gastric cancer. However, the precise mechanism has not been fully elucidated and requires subsequent research (Zhang J. et al., 2020b; Jing et al., 2021). Luo and her colleagues demonstrated that IGFBP-1 inhibited the migration of BGC-823 cells and played a protective role in *H. pylori*-induced gastric cancer, and HK and his colleagues’ data suggested that the IGF-IGFBP system may play an important role in the initiation, progression, and metastasis of gastric cancer. Since data are still lacking, future study is still urgently needed (Yi et al., 2001; Luo et al., 2017). Li et al. (2019b) elucidated that the aberrant expression of FTO has a significant prognostic value in GC patients, suggesting that FTO may play an essential role in gastric cancer progression and metastasis, and Zhou Y. et al. (2022) elucidated that FTO promotes growth and metastasis of gastric cancer *via* m6A modification of caveolin-1 and metabolic regulation of mitochondrial dynamics. Although no studies have yet elucidated the mechanism of FMR1 and RBMX in gastric cancer, many articles have demonstrated that FMR1 and RBMX were associated with the proliferation or metastasis in many kinds of cancers such as esophageal carcinoma and bladder cancer (Li et al., 2019a; Yan et al., 2021).

There are some inevitable constraints in this cohort study. First, retrospective studies may be subject to selection bias in the selection of patients. Second, due to the limited clinical information available on patients in TCGA database, more valuable clinical factors were not considered in the analysis. In addition, we did not consider other genes, particularly immune genes, which have been shown to be strongly correlated with the development and proliferation of cancer. Despite these drawbacks, our study has some notable strengths. As far as we are concerned, it is the first nomogram that integrates clinicopathological features and m6A-related genes to predict the prognosis of GC patients, which makes the predictive model more comprehensive and able to predict the survival time of each patient more accurately. Second, in addition to building the model, this study also verified the model, and the validation results showed the stability and reliability of the model.

In summary, based on the clinical and genetic data of GC patients extracted from TCGA database, we have established a relatively effective prediction model to assess the survival of GC patients at 1, 3, and 5 years, and the m6A-related genes may subsequently be targeted for the treatment of GC. The accuracy and usefulness of the nomogram still need to be further validated in subsequent clinical work.

## Data Availability

The original contributions presented in the study are included in the article/Supplementary Material; further inquiries can be directed to the corresponding author.
